# Deformable Image Registration with Inclusion of Autodetected Homologous Tissue Features

**DOI:** 10.1100/2012/913693

**Published:** 2012-04-01

**Authors:** Qingsong Zhu, Jia Gu, Yaoqin Xie

**Affiliations:** ^1^Key Laboratory for Health Informatics, Shenzhen Institutes of Advanced Technology, Chinese Academy of Sciences, Shenzhen 518055, China; ^2^Department of Radiation Oncology, Stanford University School of Medicine, Stanford, CA 94305-5847, USA

## Abstract

A novel deformable registration algorithm is proposed in the application of radiation therapy. The algorithm starts with autodetection of a number of points with distinct tissue features. The feature points are then matched by using the scale invariance features transform (SIFT) method. The associated feature point pairs are served as landmarks for the subsequent thin plate spline (TPS) interpolation. Several registration experiments using both digital phantom and clinical data demonstrate the accuracy and efficiency of the method. For the 3D phantom case, markers with error less than 2 mm are over 85% of total test markers, and it takes only 2-3 minutes for 3D feature points association. The proposed method provides a clinically practical solution and should be valuable for various image-guided radiation therapy (IGRT) applications.

## 1. Introduction

Deformable image registration is playing an increasingly important role in radiation therapy, especially in image-guided radiation therapy (IGRT). It can be used in automatic contour delineation, dose accumulation, and so forth. Many deformable image registration methods are proposed, such as B-spline models [1], finite element method (FEM) [2], optical flow [3], free-form surface-based registration [4], multiresolution optical flow technique [5], regional narrow shell model [6], and demons algorithm with the “active force” [7]. All these investigations are based on image intensity information.

On the other hand, the use of image features has been shown to substantially improve the quality of image registration [8]. For example, in landmark-based registration, feature points are manually selected and associated to construct the optimal transformation between images. Lian et al. [9] used these associated feature points to register CT and MRI images by applying thin-plate splines (TPSs) interpolation. Schreibmann and Xing [10] developed a deformable image registration algorithm in which feature points were selected manually on the template image and then detected automatically on the target image. Although the mapping is automatic, we still need to manually select feature points on the template image, which is a tedious and time-consuming process. In this work, we propose a tissue feature-based deformable algorithm with inclusion of autodetection of feature points on two images. Thousands of feature points are autodetected and automatched together, which significantly improves the accuracy and efficiency of deformable registration.

## 2. Methods

### 2.1. Software Platform

The proposed contour mapping algorithm was implemented using the Insight Toolkit (ITK) [11, 12], which is an open-source cross-platform C++ software toolkit sponsored by the National Library of Medicine (NLM). It is freely available for research purposes (http://www.itk.org/). ITK provides various basic algorithms to perform registration and segmentation for medical images. The programs contained in ITK are highly extendable, making it an ideal platform for development of image registration algorithms.

### 2.2. Overview of the Feature-Based Deformable Registration Process

The proposed feature-based deformable registration algorithm can be divided into three steps. First, feature points are selected on the template and the target image based on the gradient vectors of points in a neighborhood of each point. Then, scale-invariant features transform (SIFT) method is used to associate the corresponding feature point pairs in two images. A bidirectional mapping strategy is also applied to further increase the accuracy of feature point association. Finally, the displacement vector of an arbitrary point on the target image is obtained by interpolating the displacement vectors of the feature points pairs using TPS transformation [13].

### 2.3. Tissue Feature Association by Using SIFT Method

**Figure 1:**
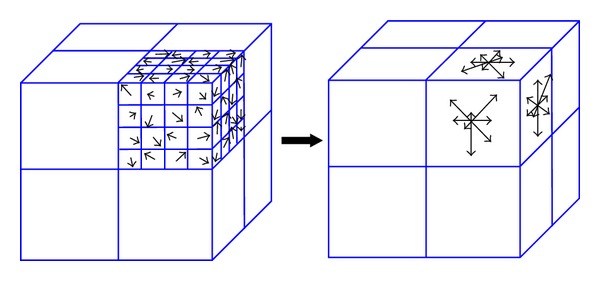
Orientation histogram of SIFT method.

Detection and association of feature points with distinct tissue feature are implemented by using SIFT method [14, 15], which uses an orientation distribution of intensity gradient vectors in eight quadrants in the neighborhood of the point (containing 8 × 8 × 8 voxels). For each quadrant (4 × 4 × 4 voxels) as illustrated in Figure 1, the gradient components in three orthogonal directions (*x*-, *y*-, *z*-axis) for each of the 64 voxels in a quadrant are computed. Let *I* and ∇*I* represent the image intensity and its intensity gradient, respectively. The gradient components along *x*-, *y*-, and *z*-axis for a voxel  (*i*, *j*, *k*)  are  (1/2)(*I*_*i*+1,*j*,*k*_ − *I*_*i*−1,*j*,*k*_), (1/2)(*I*_*i*,*j*+1,*k*_ − *I*_*i*,*j*−1,*k*_), and  (1/2)(*I*_*i*,*j*,*k*+1_ − *I*_*i*,*j*,*k*−1_), respectively. For each of the three planes (*xy*, *yz*, and *zx* planes), an eight-bin histogram of the gradient orientation with 45° interval between 0° and 360° is then constructed. Three eight-bin histograms for one of the quadrants are sketched in Figure 1. A total of 192 vectors are obtained for a given point since each quadrant has 24 vectors, with 8 vectors for each plane. The set of 192 vectors characterize the inherent features and serve as a signature of the point. The SIFT descriptor is considered as one of the most effective descriptors currently available [16]. 

For a given point, indexed by (*i*, *j*, *k*) in the template image, the least-squares difference of the SIFT descriptor of the point and its potential corresponding point  (*i*′, *j*′, *k*′)  in the target image, *S*, is computed according to

(1) $$S=\sqrt{\overset{192}{\underset{\alpha =1}{\sum }}{\left|{\left(\nabla I_{i,j,k}\right)}_{\alpha }-{\left(\nabla I_{i^{\prime },j^{\prime },k^{\prime }}\right)}_{\alpha }\right|}^{2}},$$

where *α* indexes the bins of the SIFT histogram. After the least-square difference *S* is calculated for all points in the target image, two points having the least differences *S*_1_ and *S*_2_ with point  (*i*, *j*, *k*)  in the template image are identified. If the ratio *κ* = *S*_1_/*S*_2_ is less than 50%, the corresponding point  (*i*_1_′, *j*_1_′, *k*_1_′)  with *S*_1_ is chosen as the correspondence of the point (*i*, *j*, *k*). Otherwise, no association is made to avoid any unphysical matching. More detailed discussions of the *κ*-ratio can be found in [17].

### 2.4. Bidirectional Mapping Strategy

To further increase the accuracy of feature point association, a bidirectional mapping strategy is developed based on the fact that if a point in the template image is mapped correctly to the target image, it should be default to be mapped back to the original point in the template image when an inverse map is applied to the corresponding point in the target image. Therefore, after the original association of feature points as described above, the mapped points in target image are inversely coregistered to the template image. If the correspondence still exists, the associated point pair is labeled a match. Otherwise, they are considered as a mismatch and deleted from the list of correspondence points. Upon the association of the feature points, the associated points are employed as control points.

### 2.5. TPS Deformable Transformation

The process of deformable registration is to warp the template image in such a way that it best matches the target image on a voxel-to-voxel basis. Mathematically, this is an optimization problem, in which a set of transformation parameters transform the voxels in the template image to their corresponding voxels in the target image.

To find the transformation matrix, **T**(**X**), that maps an arbitrary voxel on the template image to that on the target image (or *vice versa*), a TPS deformable model [18] is employed in this study. In brief, a weighting vector *W* = (*w*_1_, *w*_2_,…, *w*_*n*_) and the coefficients *a*_1_, *a*_*u*_, *a*_*v*_, and *a*_*w*_ are computed from a series of matrices which are constructed using *n* pairs of selected control points in the template image (*x*_*i*_, *y*_*i*_, *z*_*i*_) and the target image (*u*_*i*_, *v*_*i*_, *w*_*i*_), respectively. The function transforming a pixel coordinate in the template image to the corresponding coordinate in the target image is defined as

(2) $$\begin{array}{cc} f\left(u^{\prime },v^{\prime },w^{\prime }\right) & =a_{1}+a_{u}u+a_{v}v+a_{w}w\\ & \;+\overset{n-1}{\underset{i=0}{\sum }}w_{i}U\left(\left|p_{i}-\left(u,v,w\right)\right|\right), \end{array}$$

where *p*_*i*_ are control points coordinates in the template image, and *U* is a basis function to measure the distance. Major steps of the TPS calculation include the following.

(1) Assuming  *P*_1_ = (*x*_1_, *y*_1_, *z*_1_), *P*_2_ = (*x*_2_, *y*_2_, *z*_2_),…, *P*_*n*_ = (*x*_*n*_, *y*_*n*_, *z*_*n*_) are *n* control points in the template images, the distance between point *i* and *j* is given by *r*_*ij*_ = |*P*_*i*_ − *P*_*j*_|. Define matrices

(3) $$\begin{array}{c} P=\left[\begin{array}{cccc} 1 & x_{1} & y_{1} & z_{1}\\ 1 & x_{2} & y_{2} & z_{2}\\ \cdots & \cdots & \cdots & \cdots \\ 1 & x_{n} & y_{n} & z_{n} \end{array}\right],\\ K=\left[\begin{array}{cccc} 0 & U\left(r_{12}\right) & \cdots & U\left(r_{1n}\right)\\ U\left(r_{21}\right) & 0 & \cdots & U\left(r_{2n}\right)\\ \cdots & \cdots & \cdots & \cdots \\ U\left(r_{n1}\right) & U\left(r_{n2}\right) & \cdots & 0 \end{array}\right],\\ L=\left[\begin{array}{cc} K & P\\ P^{T} & O \end{array}\right], \end{array}$$

where *O* is a 4 × 4 matrix of zeros, and *U* is a basic function *U*(*r*) = *r* ^2^log⁡*r* ^2^.

(2) Letting  *Q*_1_ = (*u*_1_, *v*_1_, *w*_1_), *Q*_2_ = (*u*_2_, *v*_2_, *w*_2_),…, *Q*_*n*_ = (*u*_*n*_, *v*_*n*_, *w*_*n*_) be *n* corresponding control points in the target image, construct matrices

(4) $$\begin{array}{cc} V & =\left[\begin{array}{cccc} u_{1} & u_{2} & \cdots & u_{n}\\ v_{1} & v_{2} & \cdots & v_{n}\\ w_{1} & w_{2} & \cdots & w_{n} \end{array}\right],\\ Y & ={\left(\begin{array}{ccccc} V\;|\; & 0 & 0 & 0 & 0 \end{array}\right)}^{T}. \end{array}$$

The weighting vector *W* = (*w*_1_, *w*_2_,…, *w*_*n*_) and the coefficients *a*_1_, *a*_*u*_, *a*_*v*_, and *a*_*w*_ can be computed by the equation

(5) $$L^{-1}Y={\left(\begin{array}{cccc} W\;|\; & a_{1} & a_{u} & \begin{array}{cc} a_{v} & a_{w} \end{array} \end{array}\right)}^{T}.$$

(3) Using the elements of *L* ^−1^ *Y* to define a function *f*(*u*, *v*, *w*) everywhere as given in (2).

### 2.6. Evaluation of the Method Using Digital Phantom and Clinical Patient Data

The performance of the method has been evaluated by a number of 2D digital phantoms and archived clinical cases. In the digital phantom experiments, deformation was introduced by using a harmonic formula [19]

(6) $$x^{\prime }\left(x,y\right)=\left(1+b\cos mq\right)x.$$

Here, *q* = tan^−1^(*y*/*x*). Two parameters, *m* and *b*, were used to characterize a deformation. Generally, they describe the complexity and magnitude of a deformation, respectively. The accuracy of the proposed algorithm was assessed by directly comparing with the image from the known transformation matrix. A virtue of this approach is that the “ground truth” solutions exist and the transformation matrices are known, thus making the evaluation straightforward. A 3D deformable registration phantom developed by University of Michigan was also used to verify the accuracy of the algorithm.

4D CT images were acquired to test the proposed algorithm using a GE Discovery-ST CT scanner (GE Medical System, Milwaukee, WI) approximately two weeks prior to the initiation of the radiotherapy. The images were transferred through DICOM to a high-performance personal computer (PC) with a Xeon (3.6 GHz) processor for image processing. In general, quantitative validation of a deformable registration algorithm for a clinical case is difficult due to the lack of the ground truth for clinical testing cases. For the cases studied here, visual inspection method was employed to assess the success of the algorithm.

## 3. Results

### 3.1. Registration of 2D Digital Phantom

**Figure 2:**
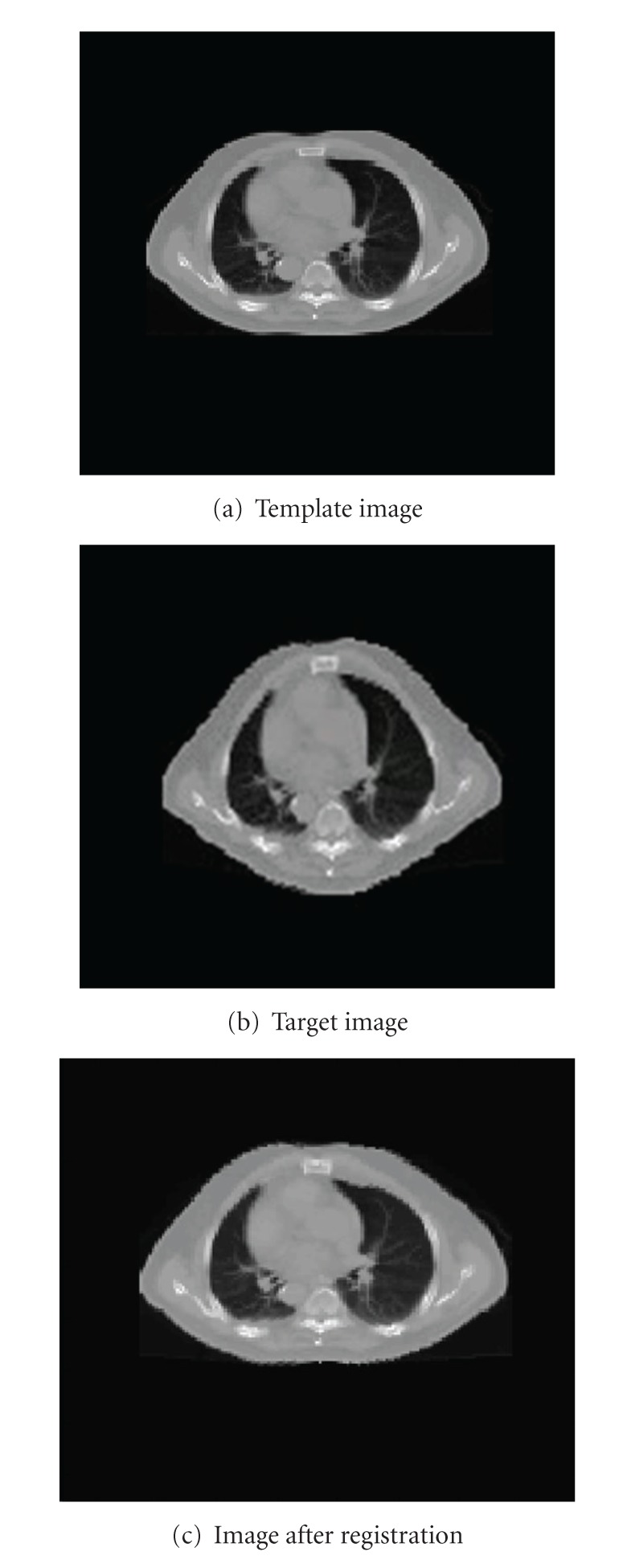
Registration result of a 2D digital phantom.

A 2D CT image (the size of the slice is 170∗170) was used in the evaluation as shown in Figure 2. The target image was generated by deforming the template image along the anterior/posterior (AP) direction using (6).

**Figure 3:**
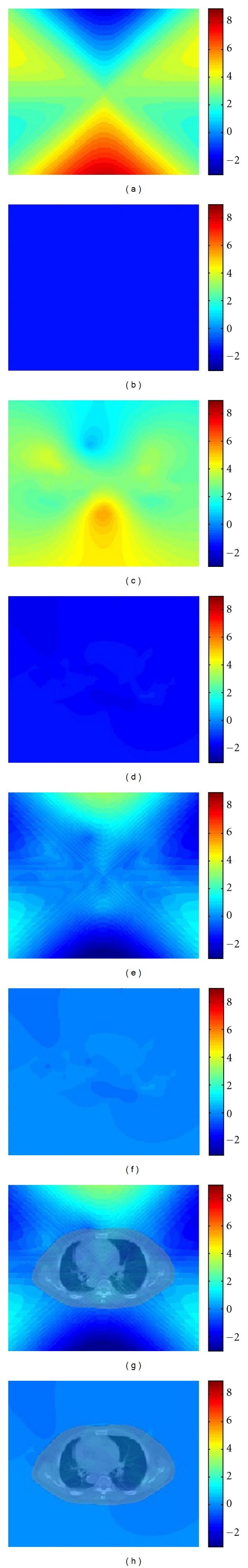
Displacement vectors fields of the 2D digital phantom.

The displacement vector field (DVF) of this experiment is shown in Figure 3. DVFs along AP direction are on the left column, and DVFs along left/right (LR) direction are on the right column. The first row is the DVF calculated by the harmonic formula as the “ground truth.” Since the deformation is along AP direction, the displacement vector along LR direction is uniform with 1.5 cm. The second row is the DVF after registration. It seems from Figures 3(a) and 3(c) that the difference between the analytical and numerical solutions is quite large. However, when the subtraction field was calculated, large errors between the analytical and numerical solutions exist only outside the phantom as shown in Figure 3(e), since no control points are outside the phantom. The largest error is about 3.0 cm. The fusion image between the subtraction field and the template image is shown in the fourth row of Figure 3. It illustrates that displacement errors are small inside the phantom.

**Figure 4:**
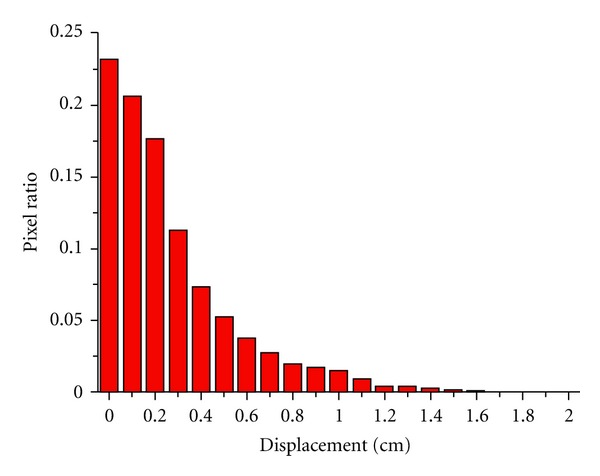
Histogram of the displacement error distribution of the 2D phantom.

Figure 4 shows the error distribution of the length of displacement vectors in the phantom case. A total of 13000 pixels were calculated; the maximum and the mean error are 1.7 cm and 0.26 cm, respectively. Errors larger than 1.0 cm exist only in 512 pixels, which is less than 4% of total pixels.

### 3.2. Registration of 3D Deformable Phantom

A 3D deformable phantom [20, 21] was used to verify the proposed method. The phantom consists of a skeleton and a lung-equivalent insert. Tumor-simulating inserts of varying density and size were embedded in the foam. The structures selected were rigid objects of known shape (balls) and various compositions. The insert was evaluated for relative attenuation using a commercial CT scanner. An actuator-driven diaphragm can compress/decompress the foam to generate 4D CT images.

**Figure 5:**
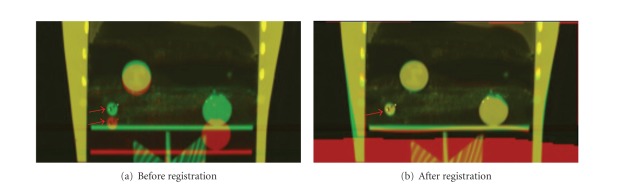
Fusion image between the inspiration and the expiration phase of a 3D deformable phantom.

Figure 5 shows the fusion images between the inspiration and the expiration phase. The inspiration phase, the expiration phase, and the overlapped region between these two phases are displayed in red, green, and yellow, respectively. Large position differences were observed before registration. The distance of a tumor (indicated by red arrows) between two phases was 1.8 cm. These differences almost disappeared after registration. 

The proposed algorithm was compared to other deformable registration methods by using the same phantom. These methods include TPS with manual selection of feature points [22], single-resolution B-splines [23], multiresolution B-splines [24–27], “demons” algorithm [7, 28], fluid flow [29, 30], and free form deformation with calculus of variations [19]. Our method was the only method that markers with error less than 2 mm are over 85% of total tested markers, and it demonstrates the high accuracy of the method [21].

### 3.3. Registration of 4D CT Images

**Figure 6:**
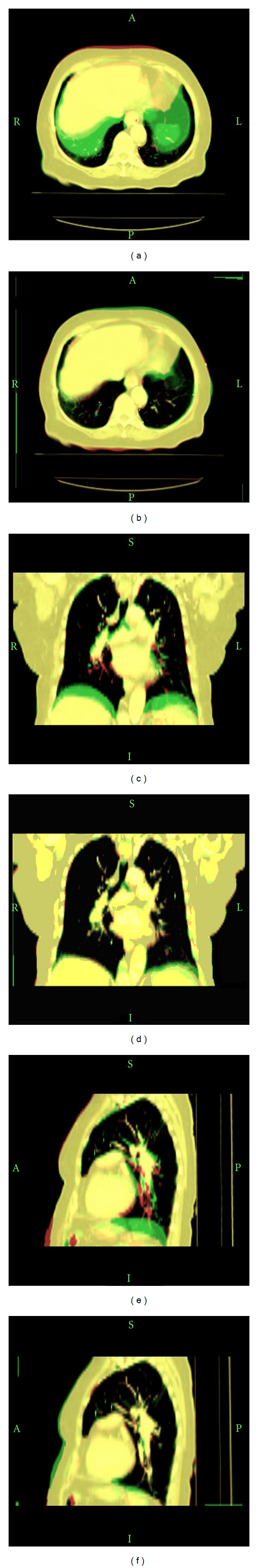
Image registration results of 4D CT images.

The proposed method was applied in clinical 4D CT images as shown in Figure 6. The fusion images before registration are on the left column, and the fusion images after registration are on the right column. The inspiration phase, the expiration phase, and the overlapped region between these two phases are displayed in red, green, and yellow, respectively. On the axial view in Figure 6(a), large difference appears in the diaphragm region, while the difference almost disappears after registration in Figure 6(b). On the frontal and sagittal view in Figures 6(c) and 6(e), the respiration movement can be clearly observed from the position change of diaphragm and trachea, while these structures are overlapped after registration in Figures 6(d) and 6(f).

**Figure 7:**
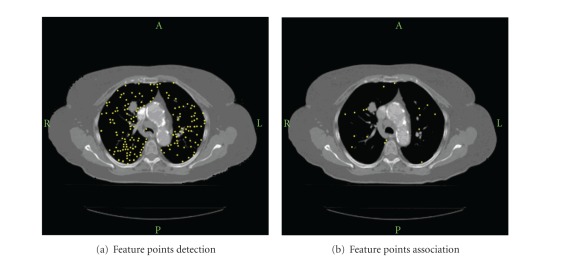
An example of feature points detection and association.

Figure 7 shows an example of feature points detection and association. 7581 feature points in the inspiration phase and 7625 in the expiration phase are detected. After the SIFT mapping, 468 control point pairs are associated, which are about 6% of the total feature points. The small percentage is due to the small *κ*-ratio of less than 50%. More detailed discussions of the *κ*-ratio can be found in [17].

## 4. Discussion

Most, if not all, registration algorithms ignore the underlying tissue features but rely on the similarity of image intensity. In contrast to intensity-based image registration, the feature-based registration extracts information regarding image structure, including shape and texture. Therefore, the feature-based image registration is generally more effective in detecting feature points. Features contained in a small control volume around a point can be used as a signature of the point [10]. The use of SIFT descriptors [15, 31, 32] is an alternative and potentially more advantageous way to associate two input images before deformable registration. The full automatic feature point detected and association make it ideally suitable for deformable registration.

It is important to address that the proposed registration method is not determined only based on the individual point information, but also based on the information about the relationship between points (e.g., continuity). In practice, although control points are discrete, the gradient vector of each point includes the continuity information.

For different purposes, control points are positioned in different regions. For instance, if we seek to separate bone and soft tissues in deformable registration, the control points are positioned in bone area. If our objective is to track respiration movement, then the control points should be positioned in the lung. Therefore, we need to define different thresholds for different tissues. For instance, the intensity threshold for bone is above 100, while for pancreas, the threshold is between 0 and 100.

Compared to B-spline deformable registration, no iterative procedure is needed in the method, and the calculation speed is at least ten times faster than B-spline registration. Commonly, it takes only 2-3 minutes for control point matching, while it may take 1 hour for B-spline registration with the same accuracy.

## 5. Conclusion

The novelties of this work include (1) seamlessly incorporation of the detected tissue feature information into accurate and robust registration and the accuracy within one voxel can be achieved; (2) without iteration procedure, the calculation speed of the proposed method can be much faster than B-spline image registration.

Inclusion of *a prior* anatomical knowledge is a key step in bringing the currently available deformable registration to the next level. The proposed method provides a clinically practical solution and should be valuable for various IGRT applications.

##  Conflict of Interests 

No actual or potential conflict of interests exists.
